# An investigation into sleep, perceived experiences, and exercise performance in elite male cyclists during the Tour de France

**DOI:** 10.14814/phy2.70395

**Published:** 2025-05-26

**Authors:** Josh Fitton, Bastien Lechat, Amy C. Reynolds, Barbara Toson, Jack Manners, Phuc Nguyen, Kelly A. Loffler, Thomas J. Altree, Peter Catcheside, Danny J. Eckert

**Affiliations:** ^1^ Flinders University, College of Medicine and Public Health, Flinders Health and Medical Research Institute – Sleep Health/Adelaide Institute for Sleep Health Adelaide South Australia Australia; ^2^ Flinders University, College of Medicine and Public Health, Flinders Clinical Trials Adelaide South Australia Australia; ^3^ Respiratory Unit The Queen Elizabeth Hospital Adelaide South Australia Australia

**Keywords:** cycling, endurance, exercise, performance, sleep

## Abstract

This study explores the dynamics of sleep, somatic/psychological experience, and exercise performance before, during, and after the Tour de France (TDF). Objective and subjective sleep, self‐reported perceived experience, and objective exercise performance data were collected daily from eight elite male cyclists across a 6‐week period including the 3‐week TDF and 11‐day pre‐ and post‐race periods. Associations between, and temporal changes in, primary interest metrics were explored through Pearson correlation and linear mixed models. Participants were (mean ± SD) aged 30 ± 4 years with overall objective sleep duration of 8 h 11mins (±58 min) per night. Sleep quality (0–100) was lower during the race than pre‐race (β [95% CI]; −8.0[−11.7, −4.3]). During the pre‐race period, sleep onset (4 [2, 5] mins) and offset times delayed (5 [3, 7] mins) and self‐reported stress increased (1.87 [1.14, 2.61]) daily. Increases in muscular soreness (0.6 [0.3, 0.8]) and fatigue (0.4 [0.2, 0.6]) during the race preceded daily declines during the post‐race period (−3.1 [−4.0, −2.1]; −2.7 [−3.5, −1.8]). Relative performance output (Performance Index; 0–1000) negatively predicted sleep duration (*r* [95% CI]; −0.32 [−0.46, −0.17]) and sleep quality (−0.34 [−0.47, −0.19]) during the race. Temporal changes in, and associations between, sleep timing, perceived experience, and exercise function highlight the potential for sleep‐improvement strategies that enhance performance in naturalistic endurance sporting contexts.

## 1 INTRODUCTION

Many psychophysiological processes important for sporting performance are impacted by, or depend upon, sleep. For example, sleep is important for physical recovery and immune function (Besedovsky et al., 2012; Mullington et al., 2010; Simpson & Dinges, 2007; Steiger, 2011), psychological processes underpinning mood, motivation, emotion regulation, and interpersonal relations (Gordon & Chen, 2014; Paterson et al., 2011); and neurocognitive and motor optimisation processes implicated in skill acquisition and learning (Lim & Dinges, 2008; Walker et al., 2002). Furthermore, sleep plays a crucial role in enhancing physical performance through muscular and cardiovascular recovery, as well as facilitation of physiological adaptation (Venter, 2012). Consequently, sleep is an important factor to consider for elite athletes seeking to optimize their sporting performance.

Athletes likely require more sleep than age‐matched counterparts to facilitate recovery from the strain of training and competition (Fullagar et al., 2015). Yet, athletes frequently sleep less than the National Sleep Foundation's recommended 7–9 h per night (Hirshkowitz et al., 2015; Sargent, Halson, & Roach, 2014; Walsh et al., 2021). Factors associated with sleep duration and quality, such as sleep onset latency, wake after sleep onset, and sleep efficiency, are often impaired in elite athletes (Gupta et al., 2017; Juliff et al., 2015; Mah et al., 2018; Swinbourne et al., 2016). This can be due to individual differences between athletes, such as competition and training schedules (Fullagar, Skorski, et al., 2016; Sargent, Lastella, et al., 2014), pre‐competition stress (Juliff et al., 2015), increasing exercise load (Killer et al., 2017; Lastella et al., 2015), and trans‐meridian or time‐sensitive travel (Fullagar, Duffield, et al., 2016; Lastella et al., 2019).

Effects of the athlete lifestyle on sleep have implications for sporting performance, particularly when athletes are engaged in endurance exercise that requires sustained aerobic output (e.g., aerobic output >30 min). Both total sleep deprivation and partial sleep restriction have been associated with poorer performance in laboratory studies on various tests of endurance performance (Bond et al., 1986; Martin, 1981; Mougin et al., 2001; Oliver et al., 2009; Skein et al., 2011), with meta‐analytic evidence suggesting larger effects from sleep deprivation than restriction (Craven et al., 2022).

The effect of impaired sleep on endurance performance is likely driven, at least in part, by the large motivational demands of sustained exercise. Sleep loss has been shown to increase ratings of perceived effort, making it more challenging to maintain the same absolute physiological output (Martin, 1981; Oliver et al., 2009; Skein et al., 2011). This underscores the importance of considering both sleep and perceived experience when evaluating endurance athletic performance.

Elite road cyclists participate in some of the most demanding endurance sport competitions and, thus, provide a unique opportunity to help understand the interplay between sleep, perceived experience, and exercise performance. The grand‐tour events, such as the Tour de France and Vuelta a España, comprise 3 weeks of consecutive race “stages”, with the majority of individual stages exceeding 150 km. The physiological demands of these events are well‐documented (Lucía et al., 2003; Sanders & van Erp, 2020; Santalla et al., 2012). Road cyclists have reported needing 8.2 h of sleep per night to feel recovered during these events (Sargent et al., 2021), but typically only achieve around 7 h per night (Lastella et al., 2015; Sargent et al., 2022, 2024).

Recent research has explored the relationship between sleep and physiological recovery during the Tour de France (Sargent et al., 2024), but the relationship between sleep and self‐reported somatic, psychological and performance experiences (e.g., mood, motivation, self‐rated performance; referred to throughout the present work as “perceived experience”) in this population remains poorly understood. Furthermore, the relationship between real‐world levels of suboptimal sleep and elite exercise performance warrants further research attention (Walsh et al., 2021). Therefore, this study was designed to explore potential associations between, and changes in, sleep, perceived experiences, and exercise performance (including readiness and stress) during a 6‐week period containing the Tour de France.

## 2 METHODS

### 2.1 Participants

Participants were eight male cyclists affiliated with a UCI (*Union Cycliste Internationale*) world‐tour cycling team. Through a formal licensing agreement, the team granted verbal and written consent to third‐party data custodians, Lumin Sports (Adelaide, South Australia), to use their de‐identified data for research purposes. Lumin Sports subsequently provided the research team with these data under an existing data‐sharing agreement. The Flinders Human Research Ethics Committee (Flinders University, South Australia) independently reviewed and approved the use of these data for the present study (project number: 5951).

**FIGURE 1:**
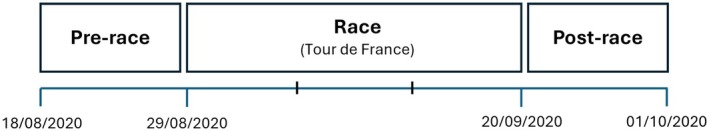
Data collection timeline across pre‐race, race, and post‐race periods. The Tour de France race period has 23 days, including two rest days on the 7th and 14th September (additional tick marks). Both pre‐ and post‐race data collection periods were 11 days.

Data were collected over an approximately 6‐week period (18th August 2020–1st October 2020) which included the 23‐day Tour de France race (21 stages; from 29th August–20th September, including two rest days on the 7th and 14th September) and 11‐day pre‐ and post‐race periods (Figure 1). Note, riders were free to consume substances (e.g., prescribed medications, alcohol, and caffeine) throughout the monitoring period that may influence sleep, perceived experience, and/or physical performance. Furthermore, ongoing medical conditions or sleep disorders may have been present. However, these data were not available to the research team.

## 3 MATERIALS

### 3.1 Objective sleep duration, sleep onset time, and sleep offset time

Garmin wristwatch devices were used to objectively estimate a range of sleep parameters through motion and heart rate signals. Parameters used in the present study were sleep duration (hours), sleep onset time, and final awake time. Garmin smartwatches are comparable to other consumer‐available health trackers for sleep estimation. Specifically, these devices demonstrate good sleep–wake classification accuracy (85%–90%), but overestimate sleep duration by ~45 min on average, compared to gold‐standard polysomnography (Chinoy et al., 2021; Miller et al., 2022; Schyvens et al., 2024). Sleep data were automatically generated by the Garmin Connect software through proprietary algorithms.

### 3.2 Exercise stress, load, and performance

Power meters integrated into the crankset of bicycles provided measures of pedal cadence and power output (Watts). Through third‐party programs (Training Peaks and Today's Plan), these parameters were used to calculate the exercise load and performance metrics that have seen widespread use in elite endurance sporting and coaching contexts. Metrics used in the present study are as follows: Performance Index (the frequency at which one is achieving relative peak power output; 0–1000 scale); Training Stress Score (exercise stress incurred relative to exercise intensity and duration, where a Training Stress Score of 100 is equivalent to 1 h spent at Functional Threshold Power); Acute Training Load (a weighted 7‐day moving average of exercise stress); Chronic Training Load (a weighted 28‐day moving average of exercise stress); and Training Stress Balance (the difference between Acute Training Load and Chronic Training Load, indicating “race readiness”). Note, a value of zero for Training Stress Balance indicates an equilibrium between training load (fitness) and recovery, and Functional Threshold Power reflects one's maximum sustainable power output for 1 h. Although the mentioned metrics relate to various exercise‐related functions, for simplicity and continuity, we will herein refer to them under the umbrella term “exercise performance” factors. Further detail on metric calculations is provided in Appendix S1. Note, only the primary bout of exercise performance data (by duration) from each day was included in analyses, to ensure only race and major training/recovery activities were used.

### 3.3 Self‐reported somatic, psychological, and performance experiences

Self‐reported experience ratings were recorded daily through the Arc Mobile (Lumin Sports) application. Self‐reports used in the present study were sleep quality, mood, fatigue, stress, motivation, soreness (muscular/physical), satisfaction, perceived performance, and feeling. These were determined using single‐item questions, such as “How fatigued are you this morning?”, answered on a visual‐analog scale on mobile devices (smartphones/tablets), with reference labels corresponding to numerical values 0–100 (e.g., “a little fatigued”; 25). Questions concerning sleep quality, fatigue, motivation, and soreness were asked at a morning check‐in; those surrounding mood, stress, feeling, perceived performance, and performance satisfaction were asked in the evening (Appendix S2). Here we refer to these self‐reported metrics collectively as “perceived experience”.

### 3.4 Statistical analyses

All statistical analyses were performed in R (version 4.2.2). We used linear mixed models (“*lme4*” R package (Bates et al., 2015)) to evaluate differences in primary interest metrics between each race period (pre‐race, race, and post‐race). Linear mixed models were chosen as they appropriately account for within‐subject correlations inherent in repeated‐measures data and imbalanced group data. Estimated marginal means derived from the modeled fixed effects were used for these planned pairwise comparisons between the different race periods. We also performed a second set of linear mixed models to explore the daily change of sleep and perceived experience outcomes within each race period: pre‐race, race, and post‐race. To achieve this, we employed an approach conceptually similar to segmented/interrupted time‐series regression methods that have seen use in quasi‐experimental, intervention‐based, research to retrospectively analyze longitudinal data (Bernal et al., 2017; Wagner et al., 2002). Such approaches allow for changes in the slope and/or level of the fitted prediction at specified points in the time‐series data: for example, the introduction of a public health initiative. Hence, an a priori decision is required on whether a change in level, slope, or both is to occur at the specified time points. Upon visual inspection of the raw data, and that large step changes in sleep and/or psychophysiological states would be unlikely to occur within a single day or night, we opted to alter slopes only. We incorporated two dummy variables into the models assessing the effect of the continuous time variable (days) on the chosen outcome. These dummy variables were coded to represent elapsed time relative to specific points in the time‐series. A first dummy variable activates at the start of the race period with the second activating at the end of the race, allowing the model to estimate separate linear slopes for the pre‐race, race, and post‐race periods. Use of these dummy variables also simplified the model, removing any need for interaction terms.

For all linear mixed models, a random intercept term per participant was included to account for repeated time‐series measurement and to control for unbalanced data and individual differences in data contribution. We did not control for common demographic variables, such as age and BMI, due to their being little variability in these factors between our eight participants. We used Pearson correlations to examine associations between sleep, perceived experience, and exercise performance during the Tour de France (race days).

All variables and models were assessed for test‐specific assumptions. We used the Benjamini‐Hochberg Procedure (Benjamini & Hochberg, 2000; Chen et al., 2017) to adjust the False Discovery Rate, reducing the likelihood of Type 1 error relative to the multiple comparisons undertaken in both the exploration of time‐dependent change and associations between the primary interest variables. An alpha level of 0.05 was employed to infer statistical significance. Figures and tables were generated in R using the base plotting function and/or the “ggplot2” (Wickham, 2011) package.

## 4 RESULTS

### 4.1 Participant information

The eight participants were all male, young‐to‐middle‐aged ([mean ± SD] 30 ± 4 years; range: 26–37 years), and had an average weight of 74 ± 14 kg. Average sleep duration across the monitoring period was 8 h and 11 min (±58 min), with mean sleep onset and wake times at 23:47 ± 00:44 (hh:mm) and 07:58 ± 00:52, respectively. Sleep duration was between 7 and 9 h on 77% of nights (pre‐race 82%; race 83%; post‐race 49%). Sleep duration of >9 h occurred on 16% of nights (pre‐race 10%; race 14%; post‐race 33%). Participants were under considerable exercise load, as evidenced by a mean Training Stress Score of 243 ± 191, reflecting approximately 2.5 h spent at Functional Threshold Power. On average, participants provided a self‐report for at least one perceived experience metric on (Mean ± SD) 85 ± 22% of all possible days (pre‐race: 96 ± 7%, race: 87 ± 28%, and post‐race 74 ± 38%). 63 ± 23% of possible days had reports for six or more of the eight perceived experience metrics (pre‐race: 66 ± 18%, race: 72 ± 27%, and post‐race: 39 ± 30%). Data for all objective sleep metrics were available on 83 ± 20% of all possible days (pre‐race: 91 ± 16%, race: 83 ± 30%, and post‐race 71 ± 38%). Performance Index was available for all participants on all possible days. The remaining performance‐related metrics (Acute Training Load, Chronic Training Load, Training Stress Balance, and Training Stress Score) were available on 50 ± 54% of possible days (pre‐race: 50 ± 54%, race: 50 ± 54%, and post‐race 43 ± 54%); 4 of the 8 participants did not have these data on any days. See Appendix S3 for descriptive statistics split by race period. All available data across the monitoring period were used in our statistical models for analyses.

### 4.2 Change in sleep, perceived experience, and exercise metrics: Pre‐race, race, and post‐race

Compared with the pre‐race period, sleep onset and wake times were delayed during the Tour de France race period. Sleep duration did not increase significantly, but subjective sleep quality declined. At post‐race, sleep duration remained stable, but sleep timing advanced, relative to the race period. Sleep timing did not significantly differ between the pre‐race and post‐race periods (Table 1).

**TABLE 1:** Inferential statistics from linear mixed models investigating differences in average sleep, perceived experience, and exercise performance between race periods (pre‐race, race, and post‐race).

Outcome variable	Difference					
	Pre‐race vs. race (*p*)		Pre‐race vs. post‐race (*p*)		Race vs. post‐race (*p*)	
Sleep						
Duration (mins)	16 [2, 18]	0.779	9 [−11, 29]	>0.999	−7 [−25, 11]	>0.999
Onset time (mins)	31 [22, 40]	**<0.001**	1 [−11, 14]	>0.999	−29 [−41, −18]	**<0.001**
Sleep offset time (mins)	47 [35, 58]	**<0.001**	10 [−5, 26]	>0.999	−36 [−50, −23]	**<0.001**
Quality (0–100)	−8.0 [−11.7, −4.3]	**<0.001**	−4.2 [−9.2, 0.8]	>0.999	8.0 [4.3, 11.8]	>0.999
Perceived experience (0–100)						
Fatigue	6.2 [3.1, 9.3]	**0.002**	−2.4 [−6.5, 1.8]	>0.999	−8.5 [−12.3, −4.8]	**<0.001**
Motivation	55.0 [50.7, 59.3]	>0.999	−9.00 [−15.3, −2.8]	0.125	−6.1 [−11.7, −0.6]	0.804
Mood	−6.0 [−9.6, −2.4]	**0.033**	−5.8 [−11.3, −0.3]	0.659	0.16 [−4.9, 5.2]	>0.999
Stress	8.7 [4.8, 12.7]	**<0.001**	−2.0 [−8.1, 4.1]	>0.999	−10.7 [−16.3, −5.1]	**0.004**
Feeling	−5.0 [−9.9, −0.2]	0.682	−13.5 [−21.0, −6.0]	**<0.012**	−8.5 [−15.2, −1.7]	0.363
Satisfaction	−8.6 [−14.5, −2.7]	0.107	−10.6 [−19.7, −1.5]	0.399	−2.0 [−10.2, 6.3]	>0.999
Soreness	12.2 [8.9, 15.5]	**<0.001**	3.0 [−1.5, 7.4]	>0.999	−9.2 [−13.1, −5.3]	**<0.001**
Performance	−8.3 [−13.3, −3.3]	**0.032**	−12.0 [−19.8, −4.2]	**0.046**	−3.7 [−10.7, 3.3]	>0.999
Exercise performance						
Performance Index	−21 [−25, −17]	**<0.001**	−33 [−39, −28]	**<0.001**	−12 [−17, 25]	**<0.001**
Acute Training Load	56 [39, 73]	**<0.001**	31 [8, 54]	0.199	−25 [−45, −4]	0.458
Chronic Training Load	12 [8, 16]	**<0.001**	20 [14, 25]	**<0.001**	8 [3.2, 13]	**0.031**
Training Stress Balance	−44 [−58, −31]	**<0.001**	−12.2 [−30.5, 6.1]	>0.999	32 [16, 48]	**0.004**
Training Stress Score	144 [83, 206]	**<0.001**	43.3 [−41.1, 127.7]	>0.999	−101 [−176, −26]	0.231

*Note*: Values are estimated Mean Differences [95% CI], *p* value. Boldfaced *p* values indicate a statistically significant effect at an alpha level of 0.05. All presented *p* values are Benjamini‐Hochberg corrected, hence values “>0.999”. Unadjusted *p* values are presented in Appendix S4. For additional context, see Appendix S3 for group descriptive statistics.

Relative to pre‐race, perceived performance and mood were lower during the race. Perceived performance was lower at post‐race (versus pre‐race). Stress, muscular soreness, and fatigue were greater during the race compared to both pre‐race and post‐race. Conversely, these metrics were not different pre‐ versus post‐race (Table 1).

Compared to pre‐race, Training Stress Score, Chronic Training Load, and Acute Training Load were greater during the race. Chronic Training Load remained elevated post‐race (versus pre‐race). Training Stress Balance and Performance Index were lower during the race (versus pre‐race), with the decrements in Performance Index persisting at post‐race (Table 1).

### 4.3 Daily change in sleep and perceived experience within pre‐race, race, and post‐race periods

**FIGURE 2:**
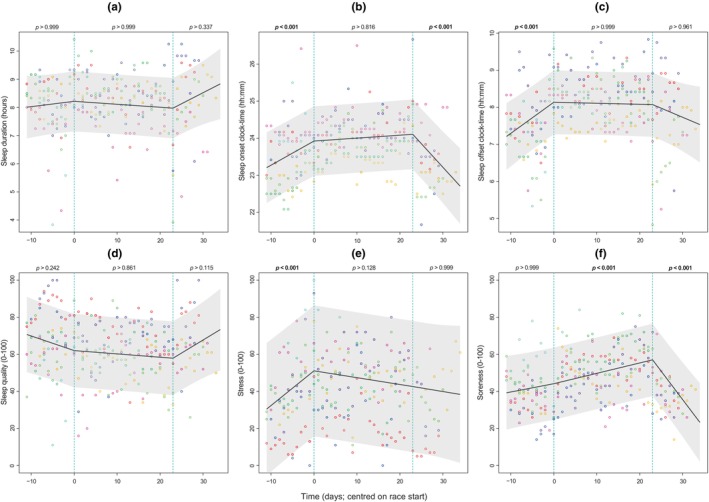
Within‐period daily change in sleep and select perceived experience outcomes: Sleep duration (a), sleep onset time (b), morning sleep offset time (c), self‐reported sleep quality (d), stress (e), and soreness (f). *p* values reflect the statistical significance of the change in the outcome variable as a function of time within the respective race period: Pre‐race, race, and post‐race. Note, the time predictor variable is centred on the start date of the Tour de France to aid interpretation. Dot points reflect raw data with unique colors for each participant. Dotted vertical lines indicate the beginning and end of the race period. Gray shade indicates 95% confidence interval boundary. Displayed *p* values are Benjamini‐Hochberg corrected.

We performed multiple linear mixed‐effect regression models to investigate daily change in sleep and perceived experience outcomes within each period (Table 2). Models with exercise performance outcomes could not be undertaken due to data missingness. Sleep onset and sleep offset times were delayed (Figure 2b,c), but sleep duration and sleep quality remained stable (Figure 2a,d) across the pre‐race period. There was no statistically significant change in any of the sleep metrics during the race period. Self‐reported stress increased progressively during the pre‐race period and remained stable thereafter during the race and post‐race (Figure 2e). During the post‐race period, sleep onset times advanced progressively. This was accompanied by no change in sleep duration or sleep quality, but notable declines in self‐reported soreness and fatigue.

**TABLE 2:** Fixed effect output from linear mixed‐effect regression investigating daily change in sleep and perceived experience outcomes within competition periods (pre‐race, race, and post‐race).

Outcome variable	Period					
	Pre‐race		Race		Post‐race	
	*β* [95% CI]	*p*	*β* [95% CI]	*p*	*β* [95% CI]	*p*
Sleep						
Duration (hh:mm)	1 [−2, 4]	>0.999	1 [−2, 1]	>0.999	5 [1, 9]	0.337
Onset time (hh:mm)	4 [2, 5]	**<0.001**	1 [0, 1]	0.816	−8 [−10, −5]	**<0.001**
Sleep offset time (hh:mm)	5 [3, 7]	**<0.001**	0 [−1, 1]	>0.999	−3 [−7, 1]	0.961
Quality (0–100)	−0.8 [−1.5, −0.1]	0.242	−0.2 [−0.4, 0.1]	0.861	1.4 [0.4, 2.5]	0.115
Perceived experience (0–100)						
Fatigue	0.2 [−0.3, 0.8]	>0.999	0.4 [0.2, 0.6]	**<0.001**	−2.7 [−3.5, −1.8]	**<0.001**
Motivation	−0.04 [−0.9, 0.8]	>0.999	−0.3 [−0.6, 0.0]	0.452	0.7 [−0.6, 2.1]	>0.999
Mood	−0.2 [−0.9, 0.4]	>0.999	−0.3 [−0.6, −0.1]	0.214	0.04 [−1.0, 1.1]	>0.999
Stress	1.9 [1.1, 2.6]	**<0.001**	−0.4 [−0.7, −0.1]	0.128	−0.4 [−1.6, 0.8]	>0.999
Feeling	−0.1 [−1.0, 0.8]	>0.999	−0.4 [−0.7, 0.0]	0.238	−0.7 [−2.1, 0.7]	>0.999
Satisfaction	−0.8 [−1.9 0.3]	>0.999	−0.2 [−0.6, 0.2]	>0.999	0.00 [−1.7, 1.7]	>0.999
Soreness	0.5 [−0.1, 1.0]	>0.999	0.6 [0.3, 0.8]	**<0.001**	−3.1 [−4.0, −2.1]	**<0.001**
Performance	−0.3 [−1.2, 0.6]	>0.999	−0.5 [−0.8, −0.1]	>0.999	0.1 [−1.3, 1.6]	>0.999

*Note*: Coefficient reflects change in outcome per day during the specified period (pre‐race, race, and post‐race). Boldfaced *p* values indicate statistical significance at an alpha level of 0.05. All presented *p* values are Benjamini‐Hochberg corrected, hence values “>0.999”. Unadjusted *p* values are presented in Appendix S5. Note that random intercept terms for participant account for potential unequal data contributions across participants.

### 4.4 Associations during the Tour de France

**FIGURE 3:**
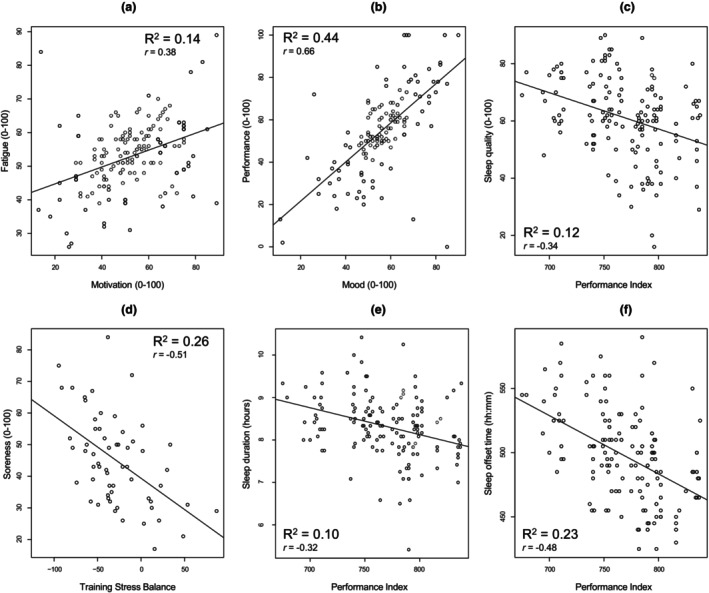
Correlation scatterplots between select variables of interest during the Tour de France race period. (a) motivation and fatigue, (b) mood and perceived performance, (c) Performance Index and sleep quality, (d) Training Stress Balance and soreness, (e) Performance Index and sleep duration, and (f) Performance Index and sleep offset time. All associations are statistically significant at *p* < 0.001 after Benjamini‐Hochberg correction.

There were multiple statistically significant associations between primary interest variables during the Tour de France period. Of particular note, there were positive associations between motivation and self‐reported fatigue (*r =* 0.38, [0.23, 0.51], *p* < 0.001; Figure 3a), and mood and perceived performance (*r =* 0.66, 95% CI [0.55, 0.74], *p* < 0.001; Figure 3b). Negative associations were present between Performance Index and sleep duration (*r* = −0.32, [−0.46, −0.17], *p* < 0.001; Figure 3e), wake time (*r* = −0.48, [−0.59, −0.34], *p* < 0.001; Figure 3f), and sleep quality (*r* = −0.34, [−0.47, −0.19], *p* < 0.001; Figure 3c). Training Stress Balance was negatively associated with self‐reported soreness (*r* = −0.51, [−0.68, −0.30], *p* < 0.001; Figure 3d). Sleep duration was negatively associated with both Acute Training Load (*r* = −0.60, [−0.74, −0.41], *p* < 0.001) and Chronic Training Load (*r* = −0.60, [−0.74, −0.40], *p* < 0.001). Correlation coefficients for all other associations between primary interest variables are provided in Appendix S6.

## 5 DISCUSSION

The present study provides novel insight into relationships between sleep, perceived experiences, and exercise performance of elite cyclists during acute pre‐race preparation, competition, and recovery from an intense multi‐day exercise event—the Tour de France. While considerable between‐subject variability was apparent, most cyclists routinely attained sleep duration within the recommended range (7–9 h). We also observed changes in sleep, perceived experience, and objective exercise load across pre‐race, race, and post‐race periods. Specifically, subjective sleep quality was reduced, and sleep timing delayed, during the race. Linear mixed models revealed changes as a function of time (days) in sleep onset time, sleep offset time, self‐reported stress, muscular soreness, and fatigue in the lead‐up to, during, and after the Tour de France.

Cyclists were routinely (77% of total nights; 83% of race nights) able to achieve sleep duration within the 7–9 h range recommended by the National Sleep Foundation (Hirshkowitz et al., 2015), with an average sleep duration (08:11 h). This aligns with the 8.2 h/night previously reported by road cyclists as being sufficient during times of heavy exercise load (Sargent et al., 2021). Furthermore, this supports previous literature that indicates that athletes from various sporting areas report sleep duration within adequate ranges (Fullagar et al., 2022; Fullagar, Skorski, et al., 2016; Lastella et al., 2015; Swinbourne et al., 2016). However, 08:11 h is >1 h more than the ~7 h sleep duration observed in a separate study of Tour de France cyclists (Sargent et al., 2024). The use of different wearable devices to estimate sleep timing (Garmin vs. WHOOP (Sargent et al., 2024)) may contribute to this discrepancy, but delayed sleep onset and wake times in our study (versus the previous Tour de France study (Sargent et al., 2024); ~1–2 h) could indicate that differences in team routine and race preparation may have been influential factors. For example, it is possible that the team in the present study preferred having daily team activities (e.g., strategic meetings) before as opposed to after competition. With race stages usually beginning between 10 am and 12 pm, and lasting approximately 4–6 h, variations in team priorities such as this could influence sleep timing. Furthermore, our data show a shift toward earlier sleep onset times during the post‐race period. Sleep duration and subjective sleep quality also appeared to increase during this period, though the effects did not reach the Benjamini‐Hochberg adjusted statistical significance threshold, potentially due to the limited available data points in the post‐race period. Tentatively, sleep timing—and perhaps sleep duration and subjective sleep quality—during the pre‐race and race periods may have been restricted and/or not sufficient for individual athletes, with post‐race rebounds occurring. These possibilities require further investigation in future studies. Although we show that it is possible for grand‐tour cyclists to routinely attain recommended sleep durations during real‐world competitive periods, post‐race “rebounds” in sleep outcomes support further research to consider that the individual sleep needs of elite athletes may not be adequately met during competition (Fullagar et al., 2015; Walsh et al., 2021).

We also observed that sleep duration remained stable, but subjective sleep quality was lower, on average during the race compared with the pre‐race period. Stability in sleep duration between race periods aligns with previous research findings in simulated and real‐world grand‐tour settings, where both a reduction or no change in sleep duration from “baseline” to race periods were observed, respectively (Lastella et al., 2015; Sargent et al., 2024). Conversely, the reduced subjective sleep quality observed in the present study conflicts with the increase in *subjective* sleep quality (from baseline) reported previously (Lastella et al., 2015). However, it should be noted that in the same study, *objective* actigraphy‐derived sleep quality declined in a similar fashion to that observed here in the present study. While fragmented sleep is typically associated with reduced sleep duration due to greater wake after sleep onset (WASO) (Bonnet & Arand, 2007), the Garmin wristwatch may not have been able to adequately detect such fragmentation (Chinoy et al., 2021; Tedesco et al., 2019). The concurrent reduction in muscular soreness and the potentially meaningful increase in subjective sleep quality within the post‐race period could indicate that increased psychophysiological load contributed to sleep disturbances during the race. The observed negative associations between sleep duration and sleep quality with Performance Index (the frequency at which riders achieve peak power output) during the race period is also consistent with this reasoning (see Figure 3). Although sleep disturbances and fragmentation might not be reflected in the Garmin‐derived estimates, they would likely manifest in the sleep quality self‐reports. Further naturalistic research, possibly leveraging ambulatory polysomnography and/or electroencephalography, would be required to further elucidate the dynamics of sleep duration and quality during real‐world endurance sporting competition.

In support of previous research that demonstrated reduced mood during periods of increased exercise load (Halson et al., 2002), we observed lower mood and self‐reported performance during the race period (versus pre‐race). Given the association between these perceived experience variables and concurrent decline, they may reflect “event‐related mood” that is influenced by variables including team dynamics, stage‐to‐stage travel, and constantly changing living and sleeping quarters with shifting accommodation during the race period. Reduced self‐reported “feeling” alongside lasting decline in self‐reported performance at post‐race could suggest a longer‐term psychological effect of the competitive period potentially akin to “burnout”. Considering that altered subjective perception, including specific factors related to burnout (Olsson et al., 2025), can impact (and potentially be impacted by) performance and team cohesion (Beedie et al., 2000; Lane et al., 2004; Lowther & Lane, 2002), our findings reinforce the importance of monitoring athletes' perceived experience during and, perhaps just as importantly, after real‐world competition.

As we cannot determine causal direction from these correlational observation, it could be that achieving peak power output at a higher frequency leads to, across the whole sample, occasions on which peak power output is met (greater performance index scores) is followed by.

Our study had several limitations. Garmin smartwatches (e.g., Vivosmart 3, Fenix 5S, and Forerunner 245) overestimate sleep duration in both the general population and athletic samples (Chinoy et al., 2021; Miller et al., 2022) when compared to gold‐standard polysomnography. This may explain, at least in part, why sleep duration in the present study was greater than reported in previous work (Sargent et al., 2024). Furthermore, Garmin “wake after sleep onset” data were not available, meaning we could not cross‐reference subjective sleep quality with objective indices (i.e., nighttime awakening frequency or sleep efficiency). Additionally, Arc Mobile questionnaire items are yet to be formally validated against their target constructs. However, their use in the present study necessarily provided low‐burden measurement that minimized disruption to team processes and, thus, maximized ecological validity. It is also possible that unknown medical history, injuries, and/or stimulant consumption (e.g., caffeine use) may have influenced some of the key study outcomes, as these data were not available to be included as covariates in the analyses. We were also unable to account for the temporal dependency between sleep and daytime activity (i.e., cycling, in this context) within the 24‐h day, which may have contributed to some of the observed negative associations between sleep (timing and duration) and performance metrics. Lastly, our niche sample limited our ability to statistically model *sleep‐dependent* change in outcomes of interest and reduces the degree to which findings can be generalized beyond the Tour de France and to other sporting populations. However, our sample is comparable to and greater than several of those used in relevant prior works (Earnest et al., 2004; Martinez‐Gonzalez, 2019; Muriel et al., 2021), and is largely unavoidable in elite sporting research, particularly during race competition.

This study offers a unique insight into elite cyclists' sleep, perceived experience, and exercise functioning during a near‐6‐week period containing the Tour de France. Notably, while there were interindividual differences, overall, riders were able to attain sleep durations within recommended ranges. Findings demonstrate temporal changes in, and associations between, sleep timing, self‐reported experience, and exercise functioning (e.g., mood, fatigue, and training stress) relative to pre‐race, race, and post‐race periods. However, further research conducted in similar real‐world sporting contexts, and using a larger sample, is warranted to more specifically test if perceived experiences and exercise functioning surrounding periods of intense endurance competition are sleep‐dependent and potentially amenable to interventions aimed at improving sleep and performance.

## AUTHOR CONTRIBUTIONS

JF, BL, ACR, and DJE developed the study concepts. JF, BL, BT, and JM contributed to data management and statistical analysis/interpretation. All authors drafted the manuscript and provided important insights into data interpretation and contributed to the final version of the manuscript. All authors approve this version of the manuscript and have agreed to the order of author presentation.

## FUNDING INFORMATION

None.

## CONFLICT OF INTEREST STATEMENT

JF reports doctoral scholarship funding from an Enterprise Scholarship arrangement between Lumin Sports and Flinders University. BL and DJE are supported by the National Health and Medical Research Council (NHMRC) of Australia Fellowships (2025886 and 1196261), and ACR is supported by the Australian Research Council (ARC). Outside the submitted work, BL has had research grants from Withings, Medical Research Future Fund, and the NHMRC. ACR declares funding from the Medical Research Future Fund, National Health and Medical Research Council, Lifetime Support Authority, Australian Research Council, Sydney Trains, Hospital Research Foundation, and Flinders Foundation unrelated to the presented work; PC reports grant funding, for trials unrelated to the presented work, from Medical Research Future Fund, National Health and Medical Research Council, Lifetime Support Authority, Motor Neurone Disease Research Australia, American Academy of Sleep Medicine, Flinders Foundation, Garnett Passe and Rodney Williams Memorial Foundation, and Invicta Medical. PC also reports equipment support for trials unrelated to the presented work from Withings, Compumedics, and Advanced Brain Monitoring. KAL has no relevant conflicts to declare. DJE has had research grants from Bayer, Apnimed, Takeda, Invicta Medical, Eli Lilly, and Withings. DJE currently serves as a scientific advisor/consultant for Apnimed, ResSleep, Invicta Medical, Takeda, and Mosanna. All other authors have no potential conflicts of interest to declare relevant to this work.

## ETHICS STATEMENT

This study was approved by the Flinders Human ResearchEthics Committee (Flinders University, South Australia) (project number: 5951).

## Supporting information


Appendix S1.

## Data Availability

Data may be made available upon reasonable request to the corresponding author, subject to agreement with data custodians Lumin Sports (Adelaide, South Australia).

## References

[phy270395-bib-0001] Bates, D. , Maechler, M. , Bolker, B. , Walker, S. , Christensen, R. H. B. , Singmann, H. , Dai, B. , Grothendieck, G. , Green, P. , & Bolker, M. B. (2015). Package ‘lme4’. Convergence, 12(1), 2.

[phy270395-bib-0002] Beedie, C. J. , Terry, P. C. , & Lane, A. M. (2000). The profile of mood states and athletic performance: Two meta‐analyses. Journal of Applied Sport Psychology, 12(1), 49–68.

[phy270395-bib-0003] Benjamini, Y. , & Hochberg, Y. (2000). On the adaptive control of the false discovery rate in multiple testing with independent statistics. Journal of Educational and Behavioral Statistics, 25(1), 60–83.

[phy270395-bib-0004] Bernal, J. L. , Cummins, S. , & Gasparrini, A. (2017). Interrupted time series regression for the evaluation of public health interventions: A tutorial. International Journal of Epidemiology, 46(1), 348–355. 27283160 10.1093/ije/dyw098 PMC5407170

[phy270395-bib-0005] Besedovsky, L. , Lange, T. , & Born, J. (2012). Sleep and immune function. Pflügers Archiv ‐ European Journal of Physiology, 463(1), 121–137. 22071480 10.1007/s00424-011-1044-0 PMC3256323

[phy270395-bib-0006] Bond, V. , Balkissoon, B. , Franks, B. , Brwnlow, R. , Caprarola, M. , Bartley, D. , & Banks, M. (1986). Effects of sleep deprivation on performance during submaximal and maximal exercise. The Journal of Sports Medicine and Physical Fitness, 26(2), 169–174. 3747483

[phy270395-bib-0007] Bonnet, M. H. , & Arand, D. L. (2007). EEG arousal norms by age. Journal of Clinical Sleep Medicine, 3(3), 271–274. 17561594 PMC2564772

[phy270395-bib-0008] Chen, S.‐Y. , Feng, Z. , & Yi, X. (2017). A general introduction to adjustment for multiple comparisons. Journal of Thoracic Disease, 9(6), 1725–1729. 28740688 10.21037/jtd.2017.05.34 PMC5506159

[phy270395-bib-0009] Chinoy, E. D. , Cuellar, J. A. , Huwa, K. E. , Jameson, J. T. , Watson, C. H. , Bessman, S. C. , Hirsch, D. A. , Cooper, A. D. , Drummond, S. P. , & Markwald, R. R. (2021). Performance of seven consumer sleep‐tracking devices compared with polysomnography. Sleep, 44(5), zsaa291. 33378539 10.1093/sleep/zsaa291 PMC8120339

[phy270395-bib-0010] Craven, J. , McCartney, D. , Desbrow, B. , Sabapathy, S. , Bellinger, P. , Roberts, L. , & Irwin, C. (2022). Effects of acute sleep loss on physical performance: A systematic and meta‐analytical review. Sports Medicine, 52(11), 2669–2690. 35708888 10.1007/s40279-022-01706-y PMC9584849

[phy270395-bib-0011] Earnest, C. P. , Jurca, R. , Church, T. , Chicharro, J. , Hoyos, J. , & Lucia, A. (2004). Relation between physical exertion and heart rate variability characteristics in professional cyclists during the tour of Spain. British Journal of Sports Medicine, 38(5), 568–575. 15388541 10.1136/bjsm.2003.005140 PMC1724921

[phy270395-bib-0012] Fullagar, H. H. , Duffield, R. , Skorski, S. , White, D. , Bloomfield, J. , Kölling, S. , & Meyer, T. (2016). Sleep, travel, and recovery responses of national footballers during and after long‐haul international air travel. International Journal of Sports Physiology and Performance, 11(1), 86–95. 10.1123/ijspp.2015-0012 25946072

[phy270395-bib-0013] Fullagar, H. H. , Skorski, S. , Duffield, R. , Hammes, D. , Coutts, A. J. , & Meyer, T. (2015). Sleep and athletic performance: The effects of sleep loss on exercise performance, and physiological and cognitive responses to exercise. Sports Medicine, 45(2), 161–186. 10.1007/s40279-014-0260-0 25315456

[phy270395-bib-0014] Fullagar, H. H. , Skorski, S. , Duffield, R. , Julian, R. , Bartlett, J. , & Meyer, T. (2016). Impaired sleep and recovery after night matches in elite football players. Journal of Sports Sciences, 34(14), 1333–1339. 10.1080/02640414.2015.1135249 26750446

[phy270395-bib-0015] Fullagar, H. H. K. , Vincent, G. E. , McCullough, M. , Halson, S. , & Fowler, P. (2022). Sleep and sport performance. Journal of Clinical Neurophysiology, 40(5), 408–416. 10.1097/WNP.0000000000000638 36930212

[phy270395-bib-0016] Gordon, A. M. , & Chen, S. (2014). The role of sleep in interpersonal conflict: Do sleepless nights mean worse fights? Social Psychological and Personality Science, 5(2), 168–175.

[phy270395-bib-0017] Gupta, L. , Morgan, K. , & Gilchrist, S. (2017). Does elite sport degrade sleep quality? A systematic review. Sports Medicine, 47, 1317–1333. 27900583 10.1007/s40279-016-0650-6 PMC5488138

[phy270395-bib-0018] Halson, S. L. , Bridge, M. W. , Meeusen, R. , Busschaert, B. , Gleeson, M. , Jones, D. A. , & Jeukendrup, A. E. (2002). Time course of performance changes and fatigue markers during intensified training in trained cyclists. Journal of Applied Physiology, 93(3), 947–956. 12183490 10.1152/japplphysiol.01164.2001

[phy270395-bib-0019] Hirshkowitz, M. , Whiton, K. , Albert, S. M. , Alessi, C. , Bruni, O. , DonCarlos, L. , Hazen, N. , Herman, J. , Katz, E. S. , & Kheirandish‐Gozal, L. (2015). National Sleep Foundation's sleep time duration recommendations: Methodology and results summary. Sleep Health, 1(1), 40–43. 10.1016/j.sleh.2014.12.010 29073412

[phy270395-bib-0020] Juliff, L. E. , Halson, S. L. , & Peiffer, J. J. (2015). Understanding sleep disturbance in athletes prior to important competitions. Journal of Science and Medicine in Sport, 18(1), 13–18. 24629327 10.1016/j.jsams.2014.02.007

[phy270395-bib-0021] Killer, S. C. , Svendsen, I. S. , Jeukendrup, A. , & Gleeson, M. (2017). Evidence of disturbed sleep and mood state in well‐trained athletes during short‐term intensified training with and without a high carbohydrate nutritional intervention. Journal of Sports Sciences, 35(14), 1402–1410. 26406911 10.1080/02640414.2015.1085589

[phy270395-bib-0022] Lane, A. M. , Terry, P. C. , Stevens, M. J. , Barney, S. , & Dinsdale, S. L. (2004). Mood responses to athletic performance in extreme environments. Journal of Sports Sciences, 22(10), 886–897. 15768723 10.1080/02640410400005875

[phy270395-bib-0023] Lastella, M. , Roach, G. D. , Halson, S. , Martin, D. , West, N. P. , & Sargent, C. (2015). The impact of a simulated grand tour on sleep, mood, and well‐being of competitive cyclists. The Journal of Sports Medicine and Physical Fitness, 55(12), 1555–1564. 25286890

[phy270395-bib-0024] Lastella, M. , Roach, G. D. , & Sargent, C. (2019). Travel fatigue and sleep/wake behaviors of professional soccer players during international competition. Sleep Health, 5(2), 141–147. 30928113 10.1016/j.sleh.2018.10.013

[phy270395-bib-0025] Lim, J. , & Dinges, D. F. (2008). Sleep deprivation and vigilant attention. Annals of the New York Academy of Sciences, 1129(1), 305–322. 18591490 10.1196/annals.1417.002

[phy270395-bib-0026] Lowther, J. , & Lane, A. (2002). Relationships between mood, cohesion and satisfaction with performance among soccer players. Athletic Insight, 4(3), 57–69.

[phy270395-bib-0027] Lucía, A. , Hoyos, J. , Santalla, A. , Earnest, C. , & Chicharro, J. L. (2003). Tour de France versus Vuelta a Espana: Which is harder? Medicine and Science in Sports and Exercise, 35(5), 872–878. 12750600 10.1249/01.MSS.0000064999.82036.B4

[phy270395-bib-0028] Mah, C. D. , Kezirian, E. J. , Marcello, B. M. , & Dement, W. C. (2018). Poor sleep quality and insufficient sleep of a collegiate student‐athlete population. Sleep Health, 4(3), 251–257. 29776619 10.1016/j.sleh.2018.02.005

[phy270395-bib-0029] Martin, B. J. (1981). Effect of sleep deprivation on tolerance of prolonged exercise. European Journal of Applied Physiology and Occupational Physiology, 47(4), 345–354. 7199438 10.1007/BF02332962

[phy270395-bib-0030] Martinez‐Gonzalez, B. (2019). The sleep of professional cyclists during a 5‐day UCI Europe tour road cycling race. Journal of Science and Cycling, 8(2), 20–22.

[phy270395-bib-0031] Miller, D. J. , Sargent, C. , & Roach, G. D. (2022). A validation of six wearable devices for estimating sleep, heart rate and heart rate variability in healthy adults. Sensors, 22(16), 6317. 36016077 10.3390/s22166317 PMC9412437

[phy270395-bib-0032] Mougin, F. , Bourdin, H. , Simon‐Rigaud, M. , Nhu, U. N. , Kantelip, J. , & Davenne, D. (2001). Hormonal responses to exercise after partial sleep deprivation and after a hypnotic drug‐induced sleep. Journal of Sports Sciences, 19(2), 89–97. 11217014 10.1080/026404101300036253

[phy270395-bib-0033] Mullington, J. M. , Simpson, N. S. , Meier‐Ewert, H. K. , & Haack, M. (2010). Sleep loss and inflammation. Best Practice & Research Clinical Endocrinology & Metabolism, 24(5), 775–784. 10.1016/j.beem.2010.08.014 21112025 PMC3548567

[phy270395-bib-0034] Muriel, X. , Valenzuela, P. L. , Mateo‐March, M. , Pallarés, J. G. , Lucia, A. , & Barranco‐Gil, D. (2021). Physical demands and performance indicators in male professional cyclists during a grand tour: WorldTour versus ProTeam category. International Journal of Sports Physiology and Performance, 17(1), 22–30. 34343966 10.1123/ijspp.2021-0082

[phy270395-bib-0035] Oliver, S. J. , Costa, R. J. , Laing, S. J. , Bilzon, J. L. , & Walsh, N. P. (2009). One night of sleep deprivation decreases treadmill endurance performance. European Journal of Applied Physiology, 107(2), 155–161. 19543909 10.1007/s00421-009-1103-9

[phy270395-bib-0036] Olsson, L. F. , Glandorf, H. L. , Black, J. F. , Jeggo, R. E. , Stanford, J. R. , Drew, K. L. , & Madigan, D. J. (2025). A multi‐sample examination of the relationship between athlete burnout and sport performance. Psychology of Sport and Exercise, 76, 102747. 39307330 10.1016/j.psychsport.2024.102747

[phy270395-bib-0037] Paterson, J. , Dorrian, J. , Ferguson, S. , Jay, S. , Lamond, N. , Murphy, P. , Campbell, S. , & Dawson, D. (2011). Changes in structural aspects of mood during 39–66 h of sleep loss using matched controls. Applied Ergonomics, 42(2), 196–201. 20659729 10.1016/j.apergo.2010.06.014

[phy270395-bib-0038] Sanders, D. , & van Erp, T. (2020). The physical demands and power profile of professional men's cycling races: An updated review. International Journal of Sports Physiology and Performance, 16(1), 3–12. 33271501 10.1123/ijspp.2020-0508

[phy270395-bib-0039] Santalla, A. , Earnest, C. P. , Marroyo, J. A. , & Lucia, A. (2012). The tour de France: An updated physiological review. International Journal of Sports Physiology and Performance, 7(3), 200–209. 22930683 10.1123/ijspp.7.3.200

[phy270395-bib-0040] Sargent, C. , Halson, S. , & Roach, G. D. (2014). Sleep or swim? Early‐morning training severely restricts the amount of sleep obtained by elite swimmers. European Journal of Sport Science, 14(sup1), S310–S315. 24444223 10.1080/17461391.2012.696711

[phy270395-bib-0041] Sargent, C. , Halson, S. L. , Martin, D. T. , & Roach, G. D. (2022). Consecutive days of racing does not affect sleep in professional road cyclists. International Journal of Sports Physiology and Performance, 17(3), 495–498. 35026733 10.1123/ijspp.2021-0102

[phy270395-bib-0042] Sargent, C. , Jasinski, S. , Capodilupo, E. R. , Powers, J. , Miller, D. J. , & Roach, G. D. (2024). The night‐time sleep and autonomic activity of male and female professional road cyclists competing in the tour de France and tour de France femmes. Sports Medicine ‐ Open, 10(1), 39. 38625486 10.1186/s40798-024-00716-6 PMC11021391

[phy270395-bib-0043] Sargent, C. , Lastella, M. , Halson, S. L. , & Roach, G. D. (2014). The impact of training schedules on the sleep and fatigue of elite athletes. Chronobiology International, 31(10), 1160–1168. 25222347 10.3109/07420528.2014.957306

[phy270395-bib-0044] Sargent, C. , Lastella, M. , Halson, S. L. , & Roach, G. D. (2021). How much sleep does an elite athlete need? International Journal of Sports Physiology and Performance, 16(12), 1746–1757. 34021090 10.1123/ijspp.2020-0896

[phy270395-bib-0045] Schyvens, A.‐M. , Van Oost, N. C. , Aerts, J.‐M. , Masci, F. , Peters, B. , Neven, A. , Dirix, H. , Wets, G. , Ross, V. , & Verbraecken, J. (2024). Accuracy of Fitbit charge 4, Garmin Vivosmart 4, and WHOOP versus polysomnography: Systematic review. JMIR mHealth and uHealth, 12(1), e52192. 10.2196/52192 38557808 PMC11004611

[phy270395-bib-0046] Simpson, N. , & Dinges, D. F. (2007). Sleep and inflammation. Nutrition Reviews, 65(suppl_3), S244–S252. 18240557 10.1111/j.1753-4887.2007.tb00371.x

[phy270395-bib-0047] Skein, M. , Duffield, R. , Edge, J. , Short, M. J. , & Mündel, T. (2011). Intermittent‐sprint performance and muscle glycogen after 30 h of sleep deprivation. Medicine & Science in Sports & Exercise, 43(7), 1301–1311. 21200339 10.1249/MSS.0b013e31820abc5a

[phy270395-bib-0048] Steiger, A. (2011). Endocrine and metabolic changes during sleep. Handbook of Clinical Neurology, 98, 241–257. 21056191 10.1016/B978-0-444-52006-7.00016-2

[phy270395-bib-0049] Swinbourne, R. , Gill, N. , Vaile, J. , & Smart, D. (2016). Prevalence of poor sleep quality, sleepiness and obstructive sleep apnoea risk factors in athletes. European Journal of Sport Science, 16(7), 850–858. 26697921 10.1080/17461391.2015.1120781

[phy270395-bib-0050] Tedesco, S. , Sica, M. , Ancillao, A. , Timmons, S. , Barton, J. , & O'Flynn, B. (2019). Validity evaluation of the Fitbit Charge2 and the Garmin vivosmart HR+ in free‐living environments in an older adult cohort. JMIR mHealth and uHealth, 7(6), e13084. 31219048 10.2196/13084 PMC6607774

[phy270395-bib-0051] Venter, R. E. (2012). Role of sleep in performance and recovery of athletes: A review article. South African Journal for Research in Sport Physical Education and Recreation, 34(1), 167–184.

[phy270395-bib-0052] Wagner, A. K. , Soumerai, S. B. , Zhang, F. , & Ross‐Degnan, D. (2002). Segmented regression analysis of interrupted time series studies in medication use research. Journal of Clinical Pharmacy and Therapeutics, 27(4), 299–309. 12174032 10.1046/j.1365-2710.2002.00430.x

[phy270395-bib-0053] Walker, M. P. , Brakefield, T. , Morgan, A. , Hobson, J. A. , & Stickgold, R. (2002). Practice with sleep makes perfect: Sleep‐dependent motor skill learning. Neuron, 35(1), 205–211. 12123620 10.1016/s0896-6273(02)00746-8

[phy270395-bib-0054] Walsh, N. P. , Halson, S. L. , Sargent, C. , Roach, G. D. , Nédélec, M. , Gupta, L. , Leeder, J. , Fullagar, H. H. , Coutts, A. J. , & Edwards, B. J. (2021). Sleep and the athlete: Narrative review and 2021 expert consensus recommendations. British Journal of Sports Medicine, 55(7), 356–368. 10.1136/bjsports-2020-102025 33144349

[phy270395-bib-0055] Wickham, H. (2011). ggplot2. Wiley Interdisciplinary Reviews: Computational Statistics, 3(2), 180–185.

