# Supplementary material for: An investigation into sleep, perceived experiences, and exercise performance in elite male cyclists during the Tour de France
Source: Physiol Rep. 2025 May 26;13(10):e70395. doi: 10.14814/phy2.70395 (PMC12106952; doi:10.14814/phy2.70395)
Supplement: Supplementary file 1 — Appendix S1. [file PHY2-13-e70395-s001.zip › PHYSREP-2025-04-316-T-s01.docx]

**An investigation into sleep, perceived experiences, and exercise performance in elite male cyclists during the Tour de France**

**Supplementary Material**

**Supplementary Material 1 |** Training and exercise functioning metrics

***Performance Index***

A metric from ‘Today’s Plan’ software, the Performance Index gauges a cyclist’s relative performance. It assesses the frequency with which a rider achieves peak power outputs over various time durations (i.e., the power curve), factors them by body weight, and combines the scores into a range of 0-1000. Performance Index provides a global estimate of performance, reflecting the rider’s recent attainment of peak power output.

***Training Stress Score (TSS)***

A metric from Training Peaks, TSS quantifies the physiological load induced by an exercise activity based on its intensity and duration. For reference, a 1-hour session undertaken at maximum sustained intensity would equate to a TSS of 100. The TSS for a 3-hour cycling activity performed at 50% intensity would be 150.

***Acute Training Load (ATL)***

Also from Training Peaks, ATL is derived from the TSS and indicates rider fatigue. The ATL score is a weighted moving average of *recent* exercise stress, calculated by adding yesterday’s ATL to the difference between Today’s TSS (exercise load) and yesterday’s ATL, divided by the time constant of 7 days.

*ATL_Today_ = ATL_Yesterday_ + (TSS_Today_ – ATL_Yesterday_) / 7*

ATL provides a marker of a rider’s physiological fatigue based on recent exercise stress.

***Chronic Training Load (CTL)***

Training Peaks suggest that their metric CTL reflects rider fitness. It is a weighted moving average of daily TSS like ATL, though over a longer period (4 weeks). It is calculated similarly to ATL but with a larger time constant.

*CTL_Today_ = CTL_Yesterday_ + (TSS_Today_ – CTL_Yesterday_) / 28*

CTL offers a historical perspective on incurred training load, deemed as accumulated fitness.

***Training Stress Balance (TSB)***

TSB, also from Training Peaks, is the difference between recent ATL and historical CTL, indicating a rider’s readiness to incur exercise stress.

*TSB = CTL_Yesterday_ – ATL_Today_*

TSB can be thought of as ‘form’ or ‘race readiness’. A neutral TSB (i.e.., 0) represents equilibrium between recent exercise stress (fatigue; ATL) and accumulated exercise stress (fitness; CTL). Positive TSB suggests a capacity to tolerate current loads (over-adaptation), whilst negative TSB indicates the opposite (under-adaptation). Training Peaks note that values below -10 indicate insufficient readiness for substantial exercise effort.

**Supplementary Material 2 |** Arc Mobile Questionnaire

Arc Mobile questionnaire items were answered on a visual-analog scale on mobile devices (smartphones/tablets), with reference labels corresponding to numerical values 0-100 (e.g., “a little fatigued”; 25). Questions concerning sleep quality, fatigue, motivation, and soreness were asked at a morning check-in; those surrounding mood, stress, feeling, performance, and satisfaction were asked in the evening.

**Morning Check-in**
***Sleep Quality:***
Question: Describe your sleep quality last night?

Labels: Severely Disturbed (0), Disturbed (25), Fair (50), Good (75), Excellent (100)

***Fatigue:***
Question: How fatigued are you this morning?

Labels: Not fatigued at all (0), A little fatigued (25), Moderately fatigued (50), Very fatigued (75), Total fatigue and exhaustion (100)

***Motivation:***
Question: What is your desire to perform physical activity today?
Labels: Extremely Low (0), Low (25), Moderate (50), High (75), Extremely High (100)

***Soreness:***
Question: How would you rate your muscular soreness?
Labels: None at all (0), Mild (33), Moderate (66), Severe (100)

**Evening Check-in**
***Mood:***
Question: Today I felt...

Labels: Extremely Negative (0), Negative (25), Indifferent (50), Positive (75), Extremely Positive (100)

***Stress:***
Question: My general level of stress today...
Labels: Extremely Low (0), Low (25), Moderate (50), High (75), Extremely High (100)

***Feeling:***
Question: During today's activity, I physically felt...

Labels: Extremely Worst Possible (0), Bad (25), Reasonable (50), Good (75), Best Possible (100)

***Performance:***
Question: My performance in today's activity was...

Labels: Extremely Worst Possible (0), Bad (25), Reasonable (50), Good (75), Best Possible (100)

***Satisfaction:***
Question: My satisfaction with today's activity was...
Labels: Extremely Low (0), Low (25), Moderate (50), High (75), Extremely High (100)

**Supplementary Material 3 |** Sample descriptive statistics for sleep, perceived experience, and exercise performance across the overall monitoring period and relative to individual race periods

|  | | | | | | | |  |
| --- | --- | --- | --- | --- | --- | --- | --- | --- |
| **Variable** | |  | **Overall** |  | **Period** | | | |
|  |  |  |  |  | ***1. Pre-Race*** | ***2. Race*** | ***3. Post-Race*** | |
| ***Sleep*** | | | | | | | |  |
|  | Duration (hh:mm) |  | 08:11 (00:58) |  | 07:57 (01:00) | 08:19 (00:44) | 08:07 (01:27) | |
|  | Onset time (hh:mm) |  | 23:47 (00:44) |  | 23:32 (00:49) | 23:58 (00:32) | 23:31 (00:58) | |
|  | Sleep offset time (hh:mm) |  | 07:58 (00:52) |  | 07:29 (00:53) | 08:17 (00:36) | 07:37 (01:07) | |
|  | Quality (0-100) |  | 63 (15) |  | 67 (18) | 61 (14) | 64 (16) | |
| ***Perceived experience (0-100)*** | | | | | | | |  |
|  | Fatigue |  | 50 (12) |  | 47 (12) | 53 (10) | 43 (13) | |
|  | Motivation |  | 52 (17) |  | 55 (17) | 52 (16) | 46 (19) | |
|  | Mood |  | 58 (13) |  | 62 (9) | 56 (14) | 56 (10) | |
|  | Stress |  | 41 (20) |  | 36 (19) | 45 (19) | 32 (19) | |
|  | Feeling |  | 56 (17) |  | 60 (12) | 56 (18) | 47 (18) | |
|  | Satisfaction |  | 56 (20) |  | 62 (13) | 54 (22) | 51 (20) | |
|  | Soreness |  | 46 (14) |  | 40 (12) | 50 (14) | 39 (12) | |
|  | Performance |  | 57 (19) |  | 63 (13) | 55 (20) | 51 (19) | |
| ***Exercise functioning*** | | | | | | | |  |
|  | Performance Index |  | 772 (40) |  | 790 (45) | 768 (37) | 761 (31) | |
|  | Acute Training Load |  | 214 (112) |  | 174 (90) | 220 (109) | 252 (138) | |
|  | Chronic Training Load |  | 207 (99) |  | 196 (99) | 199 (94) | 255 (105) | |
|  | Training Stress Balance |  | -7 (43) |  | 22 (18) | -21 (44) | 3 (40) | |
|  | Training Stress Score |  | 243 (191) |  | 153 (127) | 283 (185) | 235 (250) | |
| ***Note.*** Mean values are presented with parenthesised standard deviations. | | | | | | | |  |

**Supplementary Material 4 |** Modified version of Table 1, with unadjusted *p*-values: Inferential statistics from linear mixed models investigating change in sleep, perceived experience, and exercise functioning as a function of race period (pre-race, race, post-race).

| **Outcome Variable** | | **Effect** | | | | | |
| --- | --- | --- | --- | --- | --- | --- | --- |
|  |  | **Pre-race vs. Race** | | **Pre-race vs. Post-race** | | **Race vs. Post-race** | |
|  |  | ***β [95%CI]*** | ***p*** | ***β [95%CI]*** | ***p*** | ***β [95%CI]*** | ***p*** |
| ***Sleep*** | | | | | | | |
|  | Duration (mins) | 16 [2, 18] | **0.031** | 9 [-11, 29] | 0.368 | -7 [-25, 11] | 0.435 |
|  | Onset time (mins) | 31 [22, 40] | **<0.001** | 1[-11, 14] | 0.845 | -29 [-41, -18] | **<0.001** |
|  | Sleep offset time (mins) | 47 [35, 58] | **<0.001** | 10 [-5, 26] | 0.204 | -36 [-50, -23] | **<0.001** |
|  | Quality (0-100) | -8.0 [-11.7, -4.3] | **<0.001** | -4.2 [-9.2, 0.8] | 0.097 | 8.0 [4.3, 11.8] | 0.089 |
| ***Perceived Experience (0-100)*** | | | | | | | |
|  | Fatigue | 6.2 [3.1, 9.3] | **<0.001** | -2.4 [-6.5, 1.8] | 0.270 | -8.5 [-12.3, -4.8] | **<0.001** |
|  | Motivation | 55.0 [50.7, 59.3] | 0.221 | -9.00 [-15.3, -2.8] | **0.005** | -6.1 [-11.7, -0.6] | **0.032** |
|  | Mood | -6.0 [-9.6, -2.4] | **0.001** | -5.8 [-11.3, -0.3] | **0.039** | 0.16 [-4.9, 5.2] | 0.951 |
|  | Stress | 8.7 [4.8, 12.7] | **<0.001** | -2.0 [-8.1, 4.1] | 0.522 | -10.7 [-16.3, -5.1] | **<0.001** |
|  | Feeling | -5.0 [-9.9, -0.2] | **0.040** | -13.5 [-21.0, -6.0] | **<0.001** | -8.5 [-15.2, -1.7] | **0.014** |
|  | Satisfaction | -8.6 [-14.5, -2.7] | **0.004** | -10.6 [-19.7, -1.5] | **0.023** | -2.0 [-10.2, 6.3] | 0.642 |
|  | Soreness | 12.2 [8.9, 15.5] | **<0.001** | 3.0 [-1.5, 7.4] | 0.187 | -9.2 [-13.1, -5.3] | **<0.001** |
|  | Performance | -8.3 [-13.3, -3.3] | **0.001** | -12.0 [-19.8, -4.2] | **0.003** | -3.7 [-10.7, 3.3] | 0.300 |
| ***Exercise Functioning*** | | | | | | | |
|  | Performance Index | -21 [-25, -17] | **<0.001** | -33 [-39, -28] | **<0.001** | -12 [-17, 25] | **<0.001** |
|  | Acute Training Load | 56 [39, 73] | **<0.001** | 31 [8, 54] | **0.008** | -25 [-45, -4] | **0.018** |
|  | Chronic Training Load | 12 [8, 16] | **<0.001** | 20 [14, 25] | **<0.001** | 8 [3.2, 13] | **0.001** |
|  | Training Stress Balance | -44 [-58, -31] | **<0.001** | -12.2 [-30.5, 6.1] | 0.190 | 32 [16, 48] | **<0.001** |
|  | Training Stress Score | 144 [83, 206] | **<0.001** | 43.3 [-41.1, 127.7] | 0.312 | -101 [-176, -26] | **0.009** |
| ***Note.*** Boldfaced *p*-values indicate a statistically significant effect at an alpha level of 0.05. 95%CI = 95% confidence interval of the β regression coefficient [lower limit, upper limit]. Presented *p*-values are unadjusted. | | | | | | | |

**Supplementary Material 5 |** Modified version of Table 2, with unadjusted *p*-values: Fixed effect output from linear mixed-effect regression investigating daily change in sleep and perceived experience outcomes within competition periods (pre-race, race, post-race).

| **Outcome Variable** | | **Period** | | | | | |
| --- | --- | --- | --- | --- | --- | --- | --- |
|  |  | **Pre-race** | | **Race** | | **Post-race** | |
|  |  | ***β [95%CI]*** | ***p*** | ***β [95%CI]*** | ***p*** | ***β [95%CI]*** | ***p*** |
| ***Sleep*** | | | | | | | |
|  | Duration (mins) | 1 [-2, 4] | 0.402 | -2 [-5, 2] | 0.296 | 5 [1, 10] | **0.033** |
|  | Onset time (mins) | 4 [2, 5] | **<0.001** | -4 [-5, -1] | **0.001** | -8 [-11, -5] | **<0.001** |
|  | Sleep offset time (mins) | 5 [3, 7] | **<0.001** | -5 [-8, -2] | **<0.001** | -3 [-7, 1] | 0.187 |
|  | Quality (0-100) | -0.79 [-1.45, -0.13] | **0.019** | 0.61 [-0.21, 1.43] | 0.146 | 1.58 [0.38, 2.79] | **0.010** |
| ***Perceived Experience (0-100)*** | | | | | | | |
|  | Fatigue | 0.21 [-0.32, 0.75] | 0.432 | 0.19 [-0.48, 0.86] | 0.582 | -3.07 [-4.02, -2.11] | **<0.001** |
|  | Motivation | -0.04 [-0.88, 0.79] | 0.918 | -0.25 [-1.29, 0.79] | 0.638 | 1.02 [-0.52, 2.55] | 0.193 |
|  | Mood | -0.21 [-0.87, 0.44] | 0.523 | -0.08 [-0.91, 0.74] | 0.845 | 0.34 [-0.87, 1.55] | 0.583 |
|  | Stress | 1.87 [1.14, 2.61] | **<0.001** | -2.24 [-3.16, -1.32] | **<0.001** | -0.02 [-1.37, 1.34] | 0.979 |
|  | Feeling | -0.11 [-1.01, 0.78] | 0.803 | -0.25 [-1.36, 0.85] | 0.655 | -0.35 [-1.92, 1.21] | 0.656 |
|  | Satisfaction | -0.83 [-1.93 0.27] | 0.137 | 0.65 [-0.72, 2.01] | 0.351 | 0.21 [-1.72, 2.13] | 0.831 |
|  | Soreness | 0.45 [-0.13, 1.02] | 0.130 | 0.11 [-0.61, 0.84] | 0.757 | -3.63 [-4.69, -2.57] | **<0.001** |
|  | Performance | -0.31 [-1.24, 0.62] | 0.515 | -0.14 [-1.29, 1.01] | 0.809 | 0.57 [-1.06, 2.20] | 0.488 |
| ***Note.*** Coefficient reflects change in outcome per day during the specified period (pre-race, race, post-race). Boldfaced *p*-values indicate statistical significance at an alpha level of 0.05. Presented *p*-values are unadjusted. | | | | | | | |

**Supplementary Material 6 |** Correlation table for all possible associations between primary interest variables, with both Benjamini-Hochberg-corrected and uncorrected *p*-values. R^2^ values are also included to aid interpretation.

See attached file: “Supplementary Item 6 - Correlation_Results”
